# Presenting metabolomics analyses: what’s in a number?

**DOI:** 10.1038/s44318-024-00098-1

**Published:** 2024-04-25

**Authors:** Johannes Meiser, Christian Frezza

**Affiliations:** 1 Cancer Metabolism Group, Department of Cancer Research, Luxembourg Institute of Health, Luxembourg, Luxembourg; 2 Cluster of Excellence Cellular Stress Responses in Aging-associated Diseases (CECAD), University Hospital Cologne, Cologne, Germany; 3 Institute of Genetics, Faculty of Mathematics and Natural Sciences, Faculty of Medicine, University of Cologne, Cologne, Germany

**Keywords:** Metabolism, Methods & Resources

## Abstract

As part of a metabolism methods advice series, this commentary highlights frequent pitfalls and offers guidance related to designing, processing, and communicating metabolomics analyses.

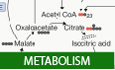

## Teaser

Metabolism analysis is complex, accordingly representation and interpretation of metabolomics and fluxomics data is prone to pitfalls, which, if not considered, can lead to misinterpretations. The purpose of this short advice article is to raise awareness for these pitfalls and provide a guideline for biologists on how to best present metabolomics data.

Pioneering work in biotechnology and engineering gave rise to the advent of metabolomics, a technology that allows the simultaneous measurement of hundreds of metabolites in a single biological specimen. This method has opened unprecedented insights into the dynamics of metabolism in health and disease, rekindling the interest in metabolism research and pushing the establishment of various metabolomics platforms. Yet, the pipeline for metabolomics analysis is very complex: from the analytical chemistry point of view, several platforms are now accessible, ranging from NMR to LC-MS, GC-MS, and more. From the data analysis perspective, the collection of large datasets often requires the aid of biostatistics and bioinformatics, methodological frameworks considered elsewhere in excellent reviews (Alseekh et al, 2021; Blaise et al, 2021; Buescher et al, 2015; Dunn et al, 2011; Ebbels et al, 2023; Jang et al, 2018; Misra, 2021). Besides these points, the representation and interpretation of metabolomics and fluxomics data is affected by several pitfalls, which, if not considered, can lead to misinterpretations. The aim of this short commentary piece is to highlight some of these pitfalls and provide a guideline for biologists on how to present metabolomics data.

## Steady-state metabolite analysis: relative versus absolute quantification

Once a metabolomics dataset has been analyzed, one of the most typical follow-up steps is to focus on altered metabolite levels. Generally, the appropriately quantified peak area of the metabolite(s) of interest is compared across conditions, and often a fold change is calculated. While useful and informative to understand whether an intervention affected a specific reaction or pathway, this approach should not be used to compare different metabolites in the same dataset to each other because of metabolite-specific analytical parameters that impact ionization efficiency. For instance, when analyzing metabolites in the TCA cycle, it is possible to infer how much fumarate is accumulated in Fumarate Hydratase-deficient cells compared to the control, but not whether fumarate is higher than succinate. Another complication could arise when analyzing the same metabolite in different biological origins (urine, plasma, tissues). In that case, the matrix effect, i.e., a difference in mass spectrometric response for a given metabolite in a standard solution or extracted from a biological matrix, can provide inaccurate quantification. An example from the cancer research field is the analysis of metabolite levels in tumor interstitial fluid compared to plasma (Sullivan et al, 2019). Even when present at the same concentration, a given metabolite will likely result in different signal intensities due to interference from the different matrices in which they are dissolved. Therefore, a side-by-side comparison could be misleading. When information about the relative abundance of a metabolite in different matrices, or the relative abundance of a metabolite against another is needed, absolute quantification of metabolite levels should be determined. This step requires a calibration curve, prepared in the same matrix in which the metabolite of interest is dissolved, and can be done either using internal or external calibration. Using a heavy isotope mixed in the sample at increasing concentration is the ideal choice, even though these labeled standards are not commercially available for many metabolites (Lu et al, 2017). Although this is cumbersome and requires additional information and careful planning of the experiment, this type of quantification is essential to evaluate if the determined concentration difference of a metabolite could have biological implications. For instance, we would know if the concentration of a metabolite lies within the K_m_ value of an enzyme that consumes it or another enzyme that it inhibits. Of note, when extrapolating this information for the absolute intracellular concentration of a metabolite, it is important to calculate the actual molarity, rather than mol × mg of tissue or million cells. In this case, besides the accurate determination of the metabolite concentration, information about the cellular volume is required.

In summary, it is important to understand the limitations of relative metabolite quantification and the challenges but also the value of absolute metabolite quantification.

## Isotope labeling to assess metabolic pathways activity

Metabolism is not a static entity but rather a highly dynamic process characterized by the constant turnover of nutrients across biochemical pathways, including the uptake and secretion of metabolites. Therefore, to assess the dynamic changes of a given metabolite or metabolic pathways, steady-state metabolomics is not sufficient, and the use of stable isotope tracers should be considered. Stable isotopes are non-radioactive variants of a given metabolite where specific elements (e.g., carbon, or nitrogen) differ in their number of neutrons, called *isotopologues*. In contrast to radioactive isotopes, which have unstable nuclei that emit radiation, stable isotopes are safe and have been routinely used in humans. All isotopologues are metabolized similarly with the advantage that downstream metabolites will contain these heavy isotopes. Notably, labeled metabolites have the same chromatographic properties as unlabeled counterparts (except in the case of deuterium, in particular in GC-MS) and will elute at the same retention time. Yet, the heavy isotope incorporation increases the mass of the molecule, and this can be measured by MS. Thus, when using a heavy labeled nutrient i.e., glucose, its metabolism can be traced throughout the intracellular metabolic network. This approach, often referred to as Stable Isotope Labeling (SIL) allows for the precise differentiation between labeled and unlabeled species, thus enabling accurate flux analysis. Several excellent reviews have been written on this topic, and we refer the readers to these for acquainting with the topic (Buescher et al, 2015; Chokkathukalam et al, 2014; Faubert et al, 2021; Hui et al, 2020; Jang et al, 2018).

### Fractional enrichment, or pool size: what should I use to present the data?

**Figure 1:**
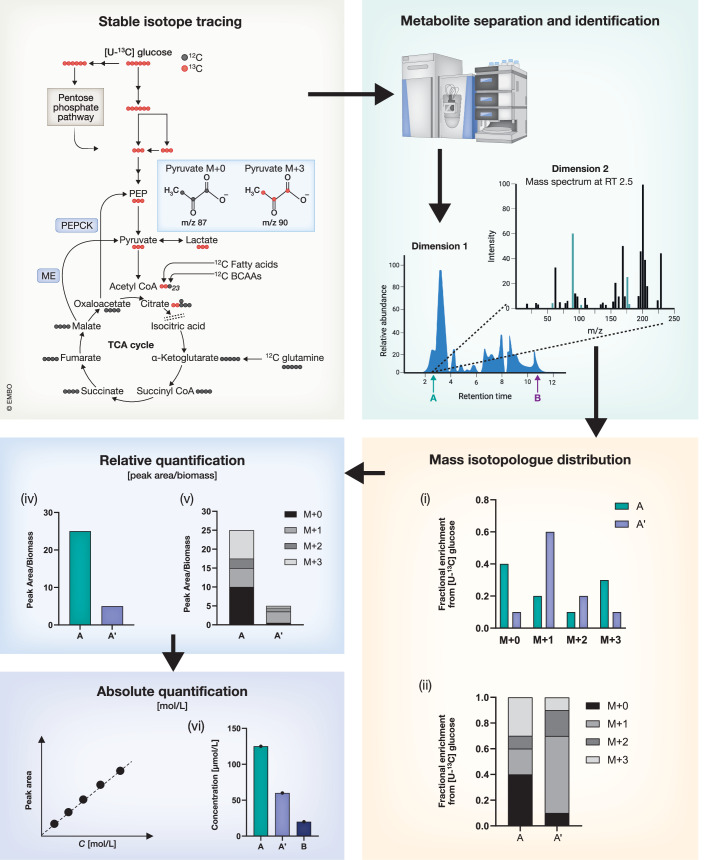
Graphical summary and experimental workflow when conducting stable isotope labeling (SIL) experiments. Gray box: Tracer selection (in this case uniformly ^13^C-labeled glucose ([U-^13^C] glucose) and its fate within the metabolic network. Importantly, contributions from other (^12^C) carbon sources can dilute or enrich the ^13^C signal in specific downstream metabolites (e.g., Acetyl-coA (the 23 indicates that Acetyl-coA has 23 carbon atoms in total, but acetyl moiety is the one eventually labeled from glucose-derived pyruvate)). The dotted lines within the TCA cycle indicate the beginning/end of one round of the cycle. Green Box: After quenching and metabolite isolation, metabolites are separated by chromatography (dimension 1) coupled to Mass Spectrometry (dimension 2). Yellow box: When all isotopologues of one metabolite are integrated, the data can be visualized as the Mass Isotopologue Distribution (MID) without the need to normalize to biomass (but after correction for natural isotope abundance). The MID shows the relative label enrichment for each isotopologue in respect to the total metabolite pool. The data can be visualized in different styles (as shown in (i) and (ii)). Blue box: The total metabolite signal (peak area of all isotopologues) can also be integrated and subsequently normalized to biomass. In this case the metabolite amount can be compared between conditions (indicated by A’) (but only for the same metabolite). When determining the absolute intracellular concentration, the concentrations of different metabolites (i.e., metabolite A and B) can be compared to each other.

When performing tracing experiments to elucidate metabolic pathway activity, it is important to discuss two important concepts: (i) pool size and (ii) the relative labeled fraction of the metabolite (Fig. 1).

The pool size of a metabolite describes the total amount of a given metabolite in a sample. However, when working with isotopes, this total amount is split into the different isotopologues of the compound. Let’s take pyruvate, the three-carbon glycolytic end-product, as an example. When cells are incubated with uniformly labeled U-¹³C_6_ glucose, the pyruvate molecule will receive three ^13^C atoms from glucose via glycolysis. If metabolized through the pentose phosphate pathway (PPP), pyruvate can also contain one or two ^13^C atoms (Antoniewicz, 2018). Of note, besides glucose, other carbon sources such as uridine-derived ribose (Nwosu et al, 2023; Skinner et al, 2023) can contribute to pyruvate and therefore to its ^13^C labeling pattern. Regardless, each incorporated ^13^C atom introduces a mass shift by one Dalton. Therefore, we can detect four different isotopologues of pyruvate: M + 0, i.e., the unlabeled, M + 1 and M + 2 from the pentose phosphate pathway (PPP), and M + 3 from glycolysis. Positional tracers e.g., glucose that only contains ^13^C atoms in positions 1 and 2 of the molecule can also be used to better resolve the respective contribution of specific pathway. In the latter case, if [1,2-^13^C_2_]glucose enters the PPP, carbon 1 of glucose will be oxidized to CO_2_ giving rise to pyruvate M + 1 labeling. If metabolized directly through glycolysis 50% of the pyruvate will be labeled M + 2 (ignoring complex cycling and recombination events through non-oxidative PPP), while the other half will be M + 0. All isotopologues elute at the same retention time, as they have identical chromatographic properties but result in separate MS peaks at distinct m/z values. Integrating each of these peaks and summing all four peak areas gives us the total pool size (=100%). The mass isotopologues distribution (MID) is the distribution of the M + *n* species of a given metabolite. When dividing a single isotopologue by the sum of all isotopologues, each isotopologue can be described as the relative labeled fraction of the total pool (expressed in percentage). MIDs are generally visualized as a stacked bar plot or an interleaved bar graph (Fig. 1). The strength of the MID is that it does not need additional normalization to biomass because it requires a relative representation. When the experiment is carried out properly, MIDs are very robust, and the data is reproducible across independent experiments. However, the limitation of MIDs is that it is without dimension, masking important information about the total pool size of a metabolite, and the absolute pathway flux. A classical analogy is given by a car park: a MID could tell us, for example, that 10% of the cars are red. However, if we do not know the size of the car park, we cannot say if that 10% equals 1, 10, or 100 red cars. Therefore, it is often informative to have both total metabolite levels (the pool size) and the relative labeling pattern. When both information is available, isotopologues can still be represented as stacked bar plots, but with their absolute values (in this case, normalized to biomass) (Fig. 1).

In addition to relative isotopologue representation, the absolute ion intensity of a selected isotopologue (normalized by its biomass) can be presented. Since there is not only production but also constant conversion of the newly built labeled molecule, caution is warranted when interpreting such data in particular at single time points. Indeed, a higher intensity of a given isotopologue could mean its increased production or lower utilization. Furthermore, when looking only at ion intensities of selected isotopologues, we do not know the absolute pool size of a metabolite. For example, selecting an isotopologue with higher abundance compared to another condition might be misleading if its absolute levels are low.

In conclusion, before plotting labeling data it is important to consider information we want to convey, and to know the limitations of each visualization method. Knowing its limits and advantages allows correct interpretation of the data to avoid misleading statements about pathway flux that are not supported by the data and underscores the importance of considering both relative and absolute measures for accurate metabolic analysis. This concept is explained in more detail in the next section (see also Boxes 1 and 2 for additional details and key considerations).

Box 1 Correction for natural abundance When working with labeling data, the first thing that needs to be done is to correct for natural isotope abundance. As the name says, natural isotopes are universally abundant in our atmosphere and interfere with our labeling data obtained from the isotope tracer. The biggest contaminant is ^13^C, which is present at 1.1% on Earth. Thus, a molecule containing 1 carbon atom has a probability of 1.1% to contain a ^13^C atom instead of a ^12^C atom. Therefore, even when running an unlabeled standard for a given molecule, it is possible to detect the M + 1 (i.e., the unlabeled metabolite plus 1 Da given by the ^13^C) and higher order isotopologues (depending on the size of the molecule, considering that a natural abundance of M + 2 will be 1.1 × 1.1%, so very low). For instance, a C4 molecule will eventually have ~ 4% of the signal as M + 1. This feature can also be used to increase the confidence of its chemical composition i.e., how many carbons it contains based on the detected natural abundance. This might seem trivial, but when looking at low enrichment data, e.g., with low pathway activity or in in vivo tracing experiments, when the contribution of a tracer is close to the natural abundance, it is easy to misinterpret the signal for actual noise. Furthermore, the bigger the molecule, the higher the natural isotope enrichment. Importantly, this natural contamination affects not only to the M + 0 molecule but to all isotopologues. For example, when palmitate, a molecule containing 16 carbons, is labeled M + 2 (this could occur when tracking palmitate synthesis from ^13^C-labeled glucose), the M + 2 isotopologue still contains 14 ^12^C atoms that can be replaced by a naturally derived ^13^C atom giving rise to M + 3 or higher isotopologues.

In conclusion, correcting for natural abundance is crucial to avoid misinterpretations (Midani et al, 2017).

Box 2 Key considerations

- When comparing a metabolite signal originating from different matrices (e.g., cell vs media, or tissue) or comparing different metabolites to each other (e.g., TCA cycle metabolites), absolute metabolite concentrations should be determined. This ensures accurate comparisons avoiding the matrix effect. This aspect is particularly important for LC-MS measurements a commonly used approach in the field.
- When performing tracing experiments, presenting both the relative labeling pattern (MID) and metabolite pool size provides a better overview of metabolic changes.
- MID is usually very robust and inferred relative flux information can already be very informative when put correctly in context.
- When performing tracing experiments, correcting for natural isotope abundance is necessary to avoid misleading data interpretation, especially for lowly abundant isotopologues.

### Caveats with interpretation of tracing experiments. An example from Acetyl-CoA labeling

It may be now helpful to clarify the term “metabolic flux”. We define flux as a conversion rate that describes the turnover of a metabolite (in mol) over time. By definition, this requires knowledge of metabolite concentration in mol. Often, reported “flux” data refer to *relative* labeling pattern assessed at single time points rather than *absolute* flux analyses. What is the difference?

This may be best explained by the fact that several nutrients often contribute to the pool of a single metabolite. Let’s take the example of glucose oxidation in the TCA cycle again (see schematic in Fig. 1). As the acetate in Acetyl-CoA contains two carbons, ^13^C tracing experiments with, e.g., fully labeled ^13^C glucose([U-^13^C] glucose) will label two carbon atoms of Acetyl-coA. M + 2 Acetyl-coA results subsequently in a citrate M + 2 enrichment (2 ^13^C atoms from Acetyl-CoA + 4 ^12^C atoms from oxaloacetate). However, the acetate moiety in Acetyl-coA is not only provided by glucose-derived pyruvate oxidation but can also come from fatty acid oxidation, glutamine catabolism (via malic enzyme) or degradation of branched-chain amino acids. Thus, when the M + 2 fraction in the citrate MID is decreased in an experiment with [U-^13^C] glucose, we can infer a decreased *relative* flux from glucose into citrate. However, reduced relative M + 2 labeling can also originate from increased fatty acid oxidation (FAO) (which contains ^12^C atoms), which would dilute the ^13^C enrichment even if the actual rate of glucose oxidation remains constant. Thus, we can say that the relative but not the absolute flux of glucose into citrate decreases. To determine the absolute flux in [mol*cell^−1^*h^−1^], we must quantify the metabolite concentration (in mol/L) and apply a dynamic tracing experiment with several time points to determine the labeling half-time (Fan et al, 2014; Meiser et al, 2016). Although this is particularly challenging for the TCA cycle, it has recently successfully been applied in vivo (Bartman et al, 2023).

Relative labeling pattern (measured at a single time point) remains very informative when comparing two conditions. Follow-up experiments could then be planned to further investigate, for example, if, indeed, pyruvate oxidation decreases or whether FAO increases (e.g., by a ^13^C palmitate tracing). To test if glutamine contributes to the pyruvate pool, a subsequent experiment can be planned with a fully labeled ^13^C glutamine tracer. Only when malic enzyme (ME) (or phosphoenolpyruvate-carboxykinase, PEPCK) is active, glutamine tracing will result in pyruvate labeling. If it is active, we can expect not only M + 4 citrate but also M + 6 citrate. The M + 6 isotopologue is representative of M + 4 oxaloacetate merged with M + 2 acetyl-coA. Thus, proper planning and interpretation of fluxomics data requires a thorough understanding of the metabolic pathways, including their atom transitions.

### Correcting for different upstream labeling pattern and different sources: when to normalize the data

One additional important aspect relates to the labeling pattern of precursor metabolites. Let’s again consider a [U-^13^C]glucose tracing experiment and subsequent tracing into the TCA cycle. Now let’s imagine that the malic enzyme, which converts malate to pyruvate, is active in condition A but not in condition B. In condition A, ^12^C from unlabeled sources (such as glutamine) will “dilute” the ^13^C glucose-derived signal in pyruvate (resulting in a lower relative M + 3 pyruvate). Consequently, M + 2 citrate labeling can be expected to be reduced in condition A, even if the rate of pyruvate oxidation remains constant. If looking only at citrate and other metabolites downstream of citrate, one may conclude that the TCA cycle activity is reduced in condition A. However, the comparison is flawed because pyruvate has already a lower labeling in condition A due to higher ME activity. To overcome this caveat, it helps to normalize the individual isotopologue data of a metabolite (in this example, citrate M + 2) to the common precursor (in this case, M + 3 pyruvate). By dividing M + 2 citrate by M + 3 pyruvate, we can normalize for labeling differences in the substrate to allow the comparison of the relative rate of pyruvate oxidation between condition A and B. Still, it has to be kept in mind that calculating these ratios, especially when the % of labeling of the metabolites involved is very low, could artificially increase the next flux of a specific reaction. This ratio-based approach becomes very practical for in vivo tracing experiments where an injected tracer is metabolized in different organs and the resulting products enter circulation alongside the tracer. In such scenario, several labeled compounds circulate in the bloodstream and can act as substrates in each tissue of interest. In the case of [U-^13^C] glucose tracing, a significant fraction of circulating M + 3 lactate will also be abundant. When analyzing the labeling pattern of TCA cycle metabolites in a tissue it is not immediately apparent whether a given ^13^C contribution is derived from ^13^C lactate or ^13^C glucose. However, by utilizing ratios this can be resolved. If glucose is the sole source of pyruvate, the ^13^C enrichment in pyruvate cannot be higher than its precursor. Therefore, a ratio between M + 3 pyruvate and its upstream metabolites must be ≤1. Only when additional carbon sources such as lactate contribute directly to the pyruvate pool, then the ratio can exceed one. Applying this clever approach, it was revealed that lactate is a meaningful substrate to tumors in vivo, challenging a dogma in the field of cancer metabolism (Faubert et al, 2017; Tasdogan et al, 2020). Parallel work combining sophisticated mathematical models with in vivo stable isotope tracing provided further insights to this observation demonstrating that circulating lactate is the predominant substrate for the TCA cycle (Hui et al, 2017).

## Metabolite consumption and release

In addition to intracellular metabolite information, metabolite exchange with the extracellular space can be relevant to probe metabolic pathway activity. In this case, it is important to clarify the difference between the simple exchange of a metabolite and its net consumption (or net release).

Let’s imagine adding ^13^C lactate to a cancer cell line cultured with high glucose (e.g., 25 mM). Due to the Crabtree effect (Crabtree, 1929), cells in culture metabolize a large fraction of glucose to lactate, which is subsequently excreted from cells into the extracellular space. Therefore, over time, the lactate concentration in the cell culture medium will increase indicating that there is a net release of lactate from the cells. However, when adding ^13^C lactate to the culture, TCA cycle metabolites will be labeled over time. In such a scenario, one could mistakenly conclude that the cells “consume” lactate. Instead, this apparent consumption is due to the exchange between the ^13^C and ^12^C pools of lactate. To illustrate this, we may remember the principles of a semi-permeable membrane. If we add an equal concentration of ^13^C lactate on one side of the membrane and ^12^C lactate on the other side, the molecules will exchange in between compartments without affecting the absolute lactate concentration on either side.

To account for these issues, cell culture medium needs to be collected at the beginning and the end of the experiment to calculate the consumption or release of each metabolite present in the culture medium. If the level of a metabolite increases over time it indicates net release and vice versa. When determining the absolute concentration, it is possible to calculate the absolute flux. By knowing the culture volume and the concentration, the absolute amount can easily be calculated and the net flux in[mol*cell^−1^*time^−1^] can be determined without complex equations. Following this approach, we could reveal that cells can release formate at comparable or even higher rates than the intracellular nucleotide synthesis flux (Meiser et al, 2016). Alternatively, we have observed a switch from glycine release to glycine consumption in cells with an activated integrated stress response (Becker et al, 2024; Ryan et al, 2021).

Finally, we would like to mention that exchange rates between in vitro and in vivo conditions can vary. For example, in recent years it has been revealed that cancer cells in vivo can also consume rather than release lactate, breaking a long-standing dogma in the field (Faubert et al, 2017; Faubert et al, 2021; Hui et al, 2017; Tasdogan et al, 2020).

## Conclusions

The field of metabolomics, including stable isotope-assisted metabolomics, has made tremendous developments during the last two decades and significantly contributed to an improved understanding of disease mechanisms. With increasing interest in related methodologies, it is important to have a profound knowledge in biochemistry and to visualize and interpret obtained data adequately to allow correct interpretation. We hope this short commentary can help raise awareness of potential pitfalls, thereby positively contributing to the development of the field.

## Glossary

Relative quantification: the measurement of the abundance of a specific metabolite within a sample relative to another reference, often a control sample. The relative quantification process involves comparing the signal intensity or peak area of the metabolite of interest in the sample of interest. The signal often requires normalization (to biomass, or internal standard) for accurate comparison.
Absolute quantification: the determination of the exact concentration of a metabolite in a sample without the need for a reference or comparison with another sample. Unlike relative quantification, absolute quantification provides precise numerical values representing the concentration of the metabolite of interest. Absolute quantification typically requires the use of calibration curves generated from known concentrations of the metabolite or the use of internal standards with known concentrations.
Matrix effect: in mass spectrometry refers to the influence of other components present in a sample matrix that can affect the efficiency of ionization and the behavior of ions within the mass spectrometer. This is a critical issue when analyzing complex samples such as biological fluids (e.g., blood, urine).
Stable isotope labeling (SIL): the incorporation of stable isotopes into metabolites of interest to analyze pathway activity. The incorporation of these substrates (usually ^13^C glucose or glutamine) results in isotopically labeled downstream metabolites that can be distinguished from their natural counterparts during mass spectrometry analysis.
Pool size: the total amount of a metabolite in a cell or tissue. In case of SIL this complete pool of metabolite can be separates in Mass Spec measurements to several isotopologues. In this case, the pool size represents the sum of all the molecules of that metabolite (isotopologues) present in a particular cellular or physiological environment.
Mass isotopologue distribution: the relative abundance pattern of isotopically labeled molecules or ions with different masses within a given metabolite. MIDs do not need further normalization to biomass since it is already relative to the total pool.
Metabolic flux: describes the turnover rate through an enzyme or pathway. A rate expresses how many moles are turned over in time. Thus, absolute metabolite concentrations are pre-requisite to calculate a flux.
Metabolite exchange and net consumption rates: the rates at which metabolites are transferred between biological compartments or consumed within a metabolic network. Many metabolites move between membranes, although the absolute concentration on each side does not need to change (metabolite exchange). If a metabolite is transported across a membrane (e.g., into a cell) with the concentration increasing on one side and decreasing on the other side, we speak about net exchange. In essence, it also describes a rate (i.e., how many moles have moved across a membrane).
